# Attitudes toward suicide among college students in South Korea and the United States

**DOI:** 10.1186/1752-4458-8-17

**Published:** 2014-05-13

**Authors:** Kristen Kim, Jong-Ik Park

**Affiliations:** 1 Department of Psychology, Princeton University, Princeton, NJ, USA; 2 Department of Psychiatry, Kangwon National University School of Medicine, Chuncheon, Republic of Korea

**Keywords:** Attitudes, Suicide, Suicide prevention, South Korea, United States

## Abstract

**Background:**

South Korea (hereafter, Korea) has witnessed a rapid increase in its suicide rate over the past few decades and currently reports the highest rate among Organization for Economic Cooperation and Development (OECD) countries. Conversely, the United States has maintained its suicide rate near the OECD average. The present study examines and compares attitudes toward suicide among college students in either country to explain the higher prevalence of suicide in Korea.

**Findings:**

Non-Korean students in the United States, Korean students in the United States, and Korean students in Korea completed a web-based questionnaire on Attitudes Toward Suicide (ATTS). A series of two-way 3 × 2 between subjects Analysis of Variance (ANOVA) tests of the participants’ group and gender, as well as post-hoc comparisons, were conducted to examine differences across various attitude domains. As expected, the results revealed group differences in the majority of attitude areas. Most notably, students in Korea reported more permissive attitudes toward suicide and were less likely to believe in the right to prevent others’ suicide. Gender did not have an effect on any attitudes except on the right to prevent suicide and there were no interactions between group and gender.

**Conclusions:**

The results suggest the importance of addressing public attitudes toward suicide in future suicide prevention efforts in Korea.

## Findings

Although most developed countries have witnessed a maintenance of or decrease in their suicide rates over recent decades, the suicide rate in South Korea (hereafter, Korea) has increased rapidly since the 1990s. Korea’s suicide rate, currently 33.5 suicides per 100,000 people, has ranked the highest among countries in the Organization for Economic Cooperation and Development (OECD) since 2003. Conversely, the United States has maintained a suicide rate of approximately 12 suicides per 100,000 people, which is close to the OECD average of 12.89 per 100,000 people [1]. Such variations in suicide rates are not wholly due to differences in living conditions, such as unemployment, divorce rates, and alcohol consumption, or in the incidence of psychiatric disorders, but instead due to sociocultural differences [2]. Public attitudes toward suicide, which reflect the sociocultural context of a population, may affect the risk of members of a population by legitimizing or inhibiting suicide or by affecting collective suicide prevention efforts. Therefore, examining underlying attitudes towards suicide may be helpful in explaining the current suicide epidemic in Korea.

Most of the existing research on attitudes toward suicide was conducted in Western countries. For example, a comparison of attitudes toward suicide among Austrian and Turkish medical students revealed differences in the students’ permissiveness toward suicide [3]. Another cross-cultural comparison of attitudes among regional politicians in Lithuania, Austria, Hungary, Norway, and Sweden revealed that politicians from countries with higher suicide rates and no national prevention strategies were more accepting of suicide than those from countries with lower suicide rates and national prevention strategies [4]. In contrast, the research on attitudes among populations in East Asia is fairly limited. One study conducted in Korea found that individuals who held more permissive attitudes toward suicide had more frequent suicidal ideation [5], but further research is necessary to clarify the role of attitudes in Korea’s suicide problem.

To address this gap in the literature, the current study examines and compares attitudes toward suicide among college students in Korea and the United States. The authors examine three different groups: Non-Korean students in the United States, Korean students in the United States, and Korean students in Korea. The main objective of this study is to identify particular attitudes that may be responsible for the prevalence of suicide in Korea. Thus, the primary hypothesis is that the students in these groups will differ in their attitudes toward suicide according to their sociocultural differences.

## Methods

A convenience sample of undergraduate students from the United States and Korea was used. Students from Princeton University in the United States and from Kangwon National University in Korea were recruited via emails containing a link to a web-based survey containing the questionnaire on Attitudes Toward Suicide (ATTS) [6]. A systematic review of the psychometric properties of 18 instruments that measure attitudes toward suicide highlighted the ATTS as the most appropriate scale for measuring attitudes toward suicide in large surveys [7]. Although it was initially developed based on college students in Sweden, it has since been utilized in various countries, such as Austria, Turkey [3], Iran [8], Nicaragua [9,10], Ghana, Uganda, and Norway [11]. It has also been used in Asian countries, such as Cambodia [9] and South Korea [5].

The original questionnaire went through a translation and back-translation between English and Korean to produce a Korean version of the questionnaire for the students in Korea [12]. Participants rated each of the 20 items on a 5-point Likert type scale (1 = *strongly disagree* to 5 = *strongly agree*). The items of the questionnaire were grouped into eight domains: permissiveness, unpredictability, incomprehensibility, noncommunication, right to prevent, preventability, relation-caused, duration of suicidal process (see Table 1).

**Table 1:** Domains and Items of the ATTS

**Domain**	**Item**
Permissiveness	“People have the right to take their own lives.”
	“There are situations in which the only reasonable solution is suicide.”
	“If someone wants to commit suicide, it is his/her own business and we should not interfere.”
	“Suicide is an acceptable means to terminate an incurable disease.”
Unpredictability	“Suicide happens without warning.”
	“Relatives usually have no idea about what is going on when a person is thinking about suicide.”
Incomprehensibility	“People who commit suicide are usually mentally ill.”
	“Suicides among younger people are particularly puzzling because they have everything to live for.”
	“Anybody can commit suicide.” (reverse)
Noncommunication	“People who talk about suicide do not necessarily commit suicide.”
	“People who make suicidal threats seldom complete suicide.”
Right to Prevent	“A suicide attempt is essentially a cry for help.”
	“It is a human duty to try to stop someone from committing suicide.”
Preventability	“It is always possible to help a person with suicidal thoughts.”
	“Once a person has made up his/her mind about committing suicide, no one can stop him/her.” (reverse)
Relation-Caused	“Most suicide attempts are caused by conflicts with a close person.”
	“Many suicide attempts are made to get revenge on or to punish someone else.”
Duration of Suicidal Process	“When a person commits suicide, it is something that he/she has considered for a long time.”
	“Most suicide attempts are impulsive actions.” (reverse)
	“Once a person has suicidal thoughts, he/she will never let them go.”

## Results

The participants were divided into three groups: (1) Non-Korean students in the United States(n = 227), (2) Korean students in the United States (n = 46), and (3) Korean students in Korea (n = 104). The mean scores and standard deviations for attitudes toward suicide by group and gender are presented in Table 2. A series of two-way 3 × 2 between subjects Analysis of Variance (ANOVA) tests with the participants’ group (Non-Korean students in the United States, Korean students in the United States, or Korean students in Korea) and gender (male, female) were performed with an alpha level of .05 to examine differences across the attitude domains.

**Table 2:** Means and standard deviations for attitudes toward suicide and suicide prevention by group and gender

		**Male**			**Female**		
	**Group**	**N**	**Mean**	**SD**	**N**	**Mean**	**SD**
Permissiveness	Non-Koreans in US	79	3.34	.860	140	3.25	.884
	Koreans in US	20	3.26	.909	24	3.76	.754
	Koreans in Korea	30	3.78	.958	68	3.60	.774
Unpredictability	Non-Koreans in US	79	3.59	.683	140	3.56	.749
	Koreans in US	20	3.43	.712	24	3.52	.801
	Koreans in Korea	30	3.27	1.03	68	3.19	.707
Incomprehensibility	Non-Koreans in US	79	3.46	.674	140	3.55	.685
	Koreans in US	20	3.31	.888	24	3.56	.650
	Koreans in Korea	30	3.52	.741	68	3.49	.772
Noncommunication	Non-Koreans in US	79	2.59	.746	140	2.67	.704
	Koreans in US	20	2.85	.829	24	2.85	.972
	Koreans in Korea	30	2.62	.652	68	2.38	.697
Right to Prevent	Non-Koreans in US	79	2.27	.816	140	2.11	.784
	Koreans in US	20	2.33	.878	24	1.71	.570
	Koreans in Korea	30	1.75	.598	68	1.61	.510
Preventability	Non-Koreans in US	79	2.20	.745	140	2.33	.747
	Koreans in US	20	1.85	.745	24	1.94	.712
	Koreans in Korea	30	2.12	.715	68	2.14	.615
Relation-Caused	Non-Koreans in US	79	3.47	.769	140	3.53	.780
	Koreans in US	20	3.70	.750	24	3.58	.670
	Koreans in Korea	30	3.67	.802	68	3.26	.725
Duration of Suicidal Process	Non-Koreans in US	79	3.08	.544	140	3.06	.520
	Koreans in US	20	2.82	.567	24	2.88	.535
	Koreans in Korea	30	3.03	.596	68	3.16	.627

Consistent with the hypothesis that the groups would differ in their attitudes toward suicide, the results revealed statistically significant main effects of group for six of the eight factors: permissiveness [*F*(2, 369) = 5.64, *p* < .01, *η*_*p*_^*2*^ = .030], unpredictability [*F*(2, 368) = 6.07, *p* < .01, *η*_*p*_^*2*^ = .032], noncommunication [*F*(2, 371) = 3.64, *p* < .05, *η*_*p*_^*2*^ = .019], right to prevent [*F*(2, 369) = 16.7, *p *< .01, *η*_*p*_^*2*^ = .083], preventability [*F*(2, 368) = 4.71, *p* < .05, *η*_*p*_^*2*^ = .025], and duration of suicidal process [*F*(2, 364) = 3.11, *p* < .05, *η*_*p*_^*2*^ = .017]. However, the results of the ANOVAs revealed that the groups did not significantly differ for incomprehensibility [*F*(2, 369) = .214, *p* > .05, *η*_*p*_^*2*^ = .001] and relation-caused [*F*(2, 370) = 1.22, *p* > .05, *η*_*p*_^*2*^ = .007]. The main effect of gender was not significant for any of the domains except for right to prevent [*F*(1, 369) = 12.12, *p* < .01, *η*_*p*_^*2*^ = .032]. The interaction of group and gender was not significant for any of the attitudes toward suicide. The results of the ANOVAs are summarized in Table 3.

**Table 3:** **Two**-**way** (**3×2**) **analysis of variance of attitudes toward suicide and suicide prevention**

**Factor**	**Source**	***SS***	***df***	***MS***	***F***	***p***	***η***_***p***_^***2***^
Permissiveness	Group	8.50	2	4.25	5.64	.004	.030
	Gender	.576	1	.576	.764	.383	.002
	Group × Gender	3.21	2	1.60	2.13	.121	.011
	Error	278	369	.754			
Unpredictability	Group	7.10	2	3.55	6.07	.003	.032
	Gender	.034	1	.034	.059	.809	.000
	Group × Gender	.701	2	.351	.599	.550	.003
	Error	215	368	.585			
Incomprehensibility	Group	.224	2	.112	.214	.808	.001
	Gender	.269	1	.269	.513	.474	.001
	Group × Gender	1.30	2	.650	1.24	.290	.007
	Error	193	369	.523			
Noncommunication	Group	4.09	2	2.04	3.64	.027	.019
	Gender	.206	1	.206	.368	.545	.001
	Group × Gender	1.96	2	.978	1.74	.177	.009
	Error	208	371	.562			
Right to Prevent	Group	18.0	2	9.00	16.7	.000	.083
	Gender	6.51	1	6.51	12.1	.001	.032
	Group × Gender	2.59	2	1.29	2.41	.092	.013
	Error	198	369	.537			
Preventability	Group	5.06	2	2.53	4.71	.010	.025
	Gender	.176	1	.176	.327	.568	.001
	Group × Gender	.225	2	.112	.209	.811	.001
	Error	198	368	.537			
Relation-Caused	Group	1.48	2	.740	1.22	.296	.007
	Gender	1.18	1	1.18	1.95	.163	.005
	Group × Gender	2.54	2	1.27	2.10	.124	.011
	Error	224	370	.606			
Duration of Suicidal Process	Group	1.97	2	.983	3.11	.046	.017
	Gender	.188	1	.188	.594	.441	.002
	Group × Gender	.286	2	.143	.453	.636	.002
	Error	115	364	.316			

Post hoc comparisons (Tukey Honestly Significant Difference Test and Games-Howell test) were used to further examine the six factors for which group had a significant main effect. For permissiveness, Korean students in Korea had a significantly higher mean than Non-Korean students in the United States, *p* = .003. For unpredictability, Non-Korean students had a significantly higher mean than Korean students in Korea, *p* < .001. For noncommunication, Korean students in the United States had a significantly higher mean than Korean students in Korea, *p* = .005. For right to prevent, Non-Korean students had a significantly higher mean than Korean students in Korea, *p* < .001. Korean students in the United States also had a significantly higher mean than Korean students in Korea, *p* = .026. For preventability, Korean students in the United States had a significantly lower mean on preventability than Non-Korean students in the United States, *p* = .004. Finally, for duration of suicidal process, Korean students in Korea had a significantly higher mean than Korean students in the United States, *p* = .029.

## Discussion

The results partially supported the main hypothesis and revealed significant group differences for six of the eight attitude domains. Students in Korea held extreme attitudes (either the highest or lowest) on five of these six domains. Post hoc comparisons elucidated that students in Korea tend to believe that suicide is permissible, the duration of suicidal process is long, suicide is predictable, people communicate their suicidal intent to others, and people do not have the right to prevent suicide. These findings suggest that students in Korea hold distinct attitudes that may relate to the startlingly high suicide rate. However, because of the complexity of the relationship between attitudes toward suicide and suicidal behavior, the specific manner in which these attitudes relate to the prevalence of suicide is unclear and the results should be interpreted with caution.

For instance, results from previous studies regarding the relationship between tolerant attitudes toward suicide and suicide risk are mixed. On the one hand, the finding that students in Korea hold more tolerant attitudes supports earlier studies suggesting that populations with greater acceptance of suicidal behavior have higher suicide rates [3,4]. However, it contradicts others relating populations with more negative and less accepting attitudes to higher suicide rates [13]. At the individual level, Colucci and Minas (2013) confirmed that higher suicide risk was associated with less negative attitudes among similarly aged students across Italy, India, and Australia. At the population level, though, they noted that more negative attitudes correlated with higher suicide rates, and proposed that this may be due to lower help-seeking tendencies in environments that are less condoning of suicide. Further investigation of this relationship on both the individual and population level is needed to make any definitive conclusions about the relationship as it exists in Korean society.

The findings that students in Korea tend to agree that the duration of suicidal process is long, suicide is predictable, and people communicate their suicidal intent to others is somewhat surprising because these indicate a belief in the possibility of suicide prevention. However, the students also reported that they believe people do not have a right to prevent suicide, which is an ethical issue. It may be that although the students believe it is *possible* to prevent suicide, they do not believe it is *ethical*, and this attitude may in turn hinder collective suicide prevention efforts. Taken together, the results suggest that if a shift in attitudes has indeed contributed to the recent rise in suicide in Korea, more permissive and less pro-prevention attitudes may be responsible.

These results must be considered in light of the limitations of this study. First, the study did not take participants’ personal exposure to suicide into account. Although previous research has shown that exposure to suicide attempts or death of others did not affect attitudes toward suicide [13], this factor may be relevant in Korea where suicide is abnormally rampant. This research is also limited in that it examines attitudes toward suicide among a non-representative, homogeneous sample of the general population and may not reflect the attitudes of the general population. Future research can expand on this study by surveying a wider population including different age groups as well as different education and socioeconomic backgrounds.

## Conclusions

Despite the limitations of the study, the findings hold valuable implications for future suicide prevention efforts in Korea. Most interestingly, students in Korea were more permissive of suicide and less likely to believe that people have the right to prevent suicide. These and other attitudes may in part explain the high prevalence of suicidal behavior and seeming ineffectiveness of prevention efforts in Korea. The results warrant further research on the role of general attitudes toward suicide on the Korean suicide problem.

## Competing interests

The authors declare that they have no competing interests.

## Authors’ contributions

KK conceived of and designed the study, acquired the data, completed the analyses and interpretation of the data, and drafted the manuscript. J-I P participated in the design and coordination of the study, assisted in data collection, and revised the manuscript. Both authors read and approved the final manuscript.
